# Quantitative Trait Locus Analysis of Seed Germination and Early Seedling Growth in Rice

**DOI:** 10.3389/fpls.2019.01582

**Published:** 2019-12-13

**Authors:** Jing Yang, Guili Yang, Meng Yang, Ling Su, Aoyun Xia, Dandan Li, Cuihong Huang, Danhua Zhou, Yongzhu Liu, Hui Wang, Zhiqiang Chen, Tao Guo

**Affiliations:** National Engineering Research Center of Plant Space Breeding, South China Agricultural University, Guangzhou, China

**Keywords:** rice, germination, early seedling growth, quantitative trait loci mapping, genetic loci

## Abstract

Seed germination and early seedling growth are important agricultural traits for developing populations of both irrigated and directly seeded rice (DSR). To investigate the genetic mechanisms underlying seed germination and early seedling growth in rice, 275 recombinant inbred lines (RILs) were genotyped in this study *via* the genotyping-by-sequencing (GBS) approach to construct a high-density linkage bin map based on the parent-independent genotyping method. Quantitative trait loci (QTLs) for 12 traits related to seed germination and early seedling growth were analyzed. Totally, 22 additive loci were detected, after analysis of the interaction between additive QTLs and environments, five stable additive loci were obtained. Among them, loci 4, 5, 12 and 14 exhibited clear pleiotropic effects that were associated with multiple traits. Analysis of the effects of the five additive stable loci showed that a single locus increased the corresponding phenotypic value. Ten of the 275 RILs pyramided the excellent alleles of the five stable genetic loci. Most phenotypic values of the ten RILs were greater than the average values. Four RILs (G260, G342, G371, and G401) with more excellent phenotypic values were subsequently selected; these RILs could serve as donor parents of favorable alleles in the breeding process. Due to the existence of pleiotropy, the use of these genetic loci for pyramid breeding can further increase the efficiency to reach breeding goals. In addition, these five stable loci have an average physical interval of only 170 kb, we also further identified five promising candidate genes by qRT-PCR, which provides us with a basis for future cloning of these genes. Overall, this work will help broaden our understanding of the genetic control of seed germination and early seedling growth, and this study provides both a good theoretical basis and a new genetic resource for the breeding of direct-seeded rice.

## Introduction

Rice (*Oryza sativa* L.) is one of the most important food crops worldwide. Recently, owing to its low cost and operational simplicity, the direct-seeding method of rice has become increasingly popular worldwide (Mahender et al., 2015). However, there are many factors limiting the growth and development of directly seeded rice (DSR), such as low emergence rates, difficulties with weeding, and population unevenness (Wang, 2009). Seed germination and seedling establishment are critical phases in rice growth and development. Seeds with rapid, uniform germination can significantly improve field emergence, and strong seedlings can improve the capability to compete against weeds at the seedling stage (Rao et al., 2007). Therefore, the mining of key genes that control seed germination and seedling establishment to elucidate their underlying molecular mechanisms is an urgent and important objective in rice breeding.

Seed germination is a complex process that can be influenced by many factors, such as seed size, genotype, seed development, storage time and environment (Sun et al., 2007; Milošević et al., 2010). To date, numerous quantitative trait loci (QTLs) for seed germination have been reported in rice (Miura et al., 2002; Fujino et al., 2004; Jiang et al., 2006; Fujino et al., 2008; Wang et al., 2010; Li et al., 2013; Liu et al., 2014; Xie et al., 2014; Cheng et al., 2015; Hsu and Tung, 2015; Kretzschmar et al., 2015; Jiang et al., 2017; Wang et al., 2017; Jin et al., 2018). However, cloned genes related to the control of rice seed germination are still rare. The first cloned related gene, *qLTG-3-1*, controls germination rate (GR) under various conditions; this gene encodes a protein with an unknown function. The expression pattern of *qLTG-3-1* suggests that its protein product may function to weaken tissues that cover the embryo during germination (Fujino et al., 2008). The second cloned related gene, *OsTPP7*, controls anaerobic germination. Under anaerobic stress, *OsTPP7* increased the turnover of trehalose-6-phosphate (T6P), thus enhancing starch mobilization and driving the growth kinetics of the germinating embryo and the elongation of the coleoptile, which consequently enhanced anaerobic germination tolerance (Kretzschmar et al., 2015). Recently, He et al. (2019) reported an isopropylmalate synthase gene and that disruption of *OsIPMS1* resulted in low seed vigor under various conditions. Further analysis confirmed that the regulation of *OsIPMS1* in rice seed vigor is involved in starch hydrolysis, glycolytic activity and energy levels in germinating seeds. Overall, these studies have provided valuable information for seed germination, but more research is still needed in this area.

Seedling vigor is controlled by many QTLs and is also one of several important agronomic traits for direct-seeding rice systems (Yano et al., 2012). During the last few decades, a large number of QTLs associated with seedling vigor have been identified (Cui et al., 2002; Zhang et al., 2005; Lu et al., 2007; Zhou et al., 2007; Cairns et al., 2009; Abe et al., 2012; Yano et al., 2012; Cheng et al., 2013; Diwan et al., 2013; Dang et al., 2014; Cordero-Lara et al., 2016; Singh et al., 2017; Zhang et al., 2017). Among these QTLs, only one major QTL for seedling height (SH) on the long arm of chromosome 3 has been finely mapped, and *OsGA20ox1* is the most likely candidate gene at this locus (Abe et al., 2012; Yano et al., 2012). Therefore, mapping more QTLs and identifying effective causal genes is necessary.

Currently, most of the reported QTLs identified for seed germination and seedling vigor have been based on traditional markers such as simple sequence repeats (SSRs) and restricted fragment length polymorphisms (RFLPs). High-density single-nucleotide polymorphisms (SNPs) are becoming more readily available owing to the development of sequencing technology, and the genomic information that they provide is expected to bridge the gap between QTLs and candidate genes (Mao et al., 2015; Kooke et al., 2016; Si et al., 2016).

In this study, we grew 275 recombinant inbred lines (RILs) derived from two *indica* parents and collected data concerning 12 traits; these data were then used to comprehensively evaluate the seed germination and seedling establishment ability of rice. In addition, we applied the genotyping-by-sequencing (GBS) approach to sequence the 275 RILs and constructed a high-density linkage bin map based on the method of parent-independent genotyping (Xie et al., 2010). In total, 42 QTLs were detected, which will provide an improved understanding of the genetic basis for seed germination and early seedling growth. In particular, these QTLs provide a promising target for marker-assisted breeding for DSR varieties.

## Materials and Methods

### Plant Materials

We constructed a RIL population consisting of 275 lines derived from two *indica* parents, H335 and CHA-1 by the seed single descent method. The RILs were grown in a paddy field at South China Agricultural University, Guangzhou, China (at approximately 113° east longitude and approximately 23° north latitude), during the wet season (WS; F_6_) and dry season (DS; F_7_) in 2017. Each RIL or parent was planted in a block design (6 columns × 6 rows), with a spacing of 20 cm between plants. Crop management and disease and insect pest control were performed in accordance with location recommendations. All the materials were obtained from the germplasm resource bank of the National Engineering Research Center of Plant Space Breeding. Considering the effects of seed maturity, six individual plants in the middle of each block were harvested independently on the 35th day after heading in the WS and on the 40th day after heading in the DS. The harvested seeds were dried in a heated air dryer at 42°C for 5 days and then stored at −20°C.

### Germination Assessment and Evaluation of Seedling Vigor-Related Traits

Seeds from three plants were selected, placed in a 50°C oven, and treated with dry heat for 7 days to break dormancy. All seeds were surface sterilized with 20% diluted bleach (6–7% NaClO) for 20 min and then rinsed three times with sterile distilled water. To analyze seed germination, 100∼200 seeds (two replicates per plant) were randomly placed in a Petri dish and covered with two layers of 9 cm circular pieces of filter paper, after which 10 ml of sterilized distilled water was added. All Petri dishes were placed in a chamber that had an 8 h light (200 µmol m^-2^ s^-1^)/16 h dark photoperiod at 30°C. The seeds were considered to have germinated when their radicle or germ length reached approximately 1 mm. The germinability of the seeds was observed daily to calculate the germination percentage. The percentage of germinated seeds after 3 days was referred to as the germination potential (GP), and the percentage of germinated seeds after 7 days was referred to as the final GR. The germination index (GI) was calculated by the method of Wang et al. (2010) as follows: GI  =  ∑*Gt/t*, where *Gt* is the number of germinated seeds on day *t*. Ten seedlings per dish were randomly selected after 7 days of germination, and the SH was measured with a standard ruler. The seed vitality index (VI) was then calculated as follows: VI = GI*S, where GI is the germination index and S is the average height of the seedlings.

To measure early seedling vigor, we followed the abovementioned method used for pregermination. Three plants were selected per RIL. Ten seeds (whose radicle or germ length reached approximately 1 mm) per individual were selected, placed in a germination box (length of 19 cm, width of 13 cm, height of 12 cm) and covered with two layers of filter paper, after which 20 ml of sterilized distilled water was added. The culture conditions were the same as those listed above. After 7 days, a WinRHIZO (Regent Instruments Inc., Québec, Canada) root image analysis system was used to measure the seedling stem diameter (SSD), root length (RL), root surface area (RSA), root volume (RV), and root diameter (RD). Root fresh weight (RFW) and shoot fresh weight (SFW) were measured by a sensitive balance, and SH was measured using a standard ruler.

### Statistical Analyses

Broad-sense heritability (*H* *^2^*) of traits across multiple environments was calculated according to the methods of Knapp et al. (1985). Heritability was calculated as *H* *^2^* = *δ* *^2^* *_g_*/(*δ* *^2^* *_g_* + *δ* *^2^* *_ge_*/*e* + *δ* *^2^*/*e* × *r*), where *δ* *^2^* *_g_* is the genetic variance, *δ* *^2^* *_ge_* is genotype × environment interaction, *δ* *^2^* is the error variance, *e* is the number of environments, and *r* is the number of replications per environment. Statistical analysis was performed with Statistical Analysis System (SAS, version 8.01) and Microsoft Excel. Calculate variance with QTL IciMapping v4.1 software (Meng et al., 2015).

### QTL Analyses

Young leaves were collected from the seedlings of 275 RILs (F_6_), and their genomic DNA was extracted using the cetyl-trimethylammonium bromide (CTAB) method (Healey et al., 2014). SNP calling and the bin map construction method were based on those in a previous report (Huang et al., 2009; Xie et al., 2010). Genotypes were called based on SNP ratios. The breakpoints were determined at the boundary of the different genotypes, and bin markers were obtained by combining genotypes with recombination breakpoints. The resequencing data of RILs the following linkage maps data were not shown. QTL IciMapping v4.1 software (Meng et al., 2015) was used for the QTL analyses. Additive QTLs were mapped using the inclusive composite interval mapping of additive (ICIM-ADD) mapping method. The threshold for the logarithm of odds (LOD) scores was set to 2.5. For QTL × environment interactive effects, the threshold for LOD scores was set to 2.5. For epistatic QTLs, *via* the inclusive composite interval mapping of epistatic (ICIM-EPI) mapping method, the threshold for LOD scores was set to 5.0. To determine whether segregation distortion of markers occurred in the QTLs detected in this study, interval mapping of segregation distortion locus (SDLs) was also conducted. Significant SDLs were declared for loci exceeding the 2.5 LOD threshold level.

### Validation of Candidate Genes by Real-Time Quantitative Reverse Transcriptase Polymerase Chain Reaction

The total RNA of each sample was homogenized using a mortar and pestle with liquid nitrogen and then purified using a Plant Total RNA Purification Kit (ComWin Biotech Company) following the manufacturer’s instructions. The RNA samples were reverse transcribed into cDNA using a high-capacity cDNA archive kit (Applied Biosystems, USA). Quantitative Reverse Transcriptase Polymerase Chain Reaction (qRT-PCR) was then conducted using AceQ qPCR SYBR Green Master Mix Kit (Vazyme Biotech) according to the standard protocol, and the expression levels of the genes were determined on a StepOnePlus System (Applied Biosystems, USA). Three replicates were included for each treatment. As an endogenous control, Actin was used for the normalization of the cycle threshold (Ct) value obtained, and the relative expression values were calculated by the ΔΔCt method. Gene-specific primers were designed using NCBI primer BLAST (http://www.ncbi.nlm.nih.gov/tools/primer-blast/), and the primer sequences of the 21 candidate genes are listed in **Supplementary Table S1**.

## Results

### Phenotypic Variation

**Figure 1:**
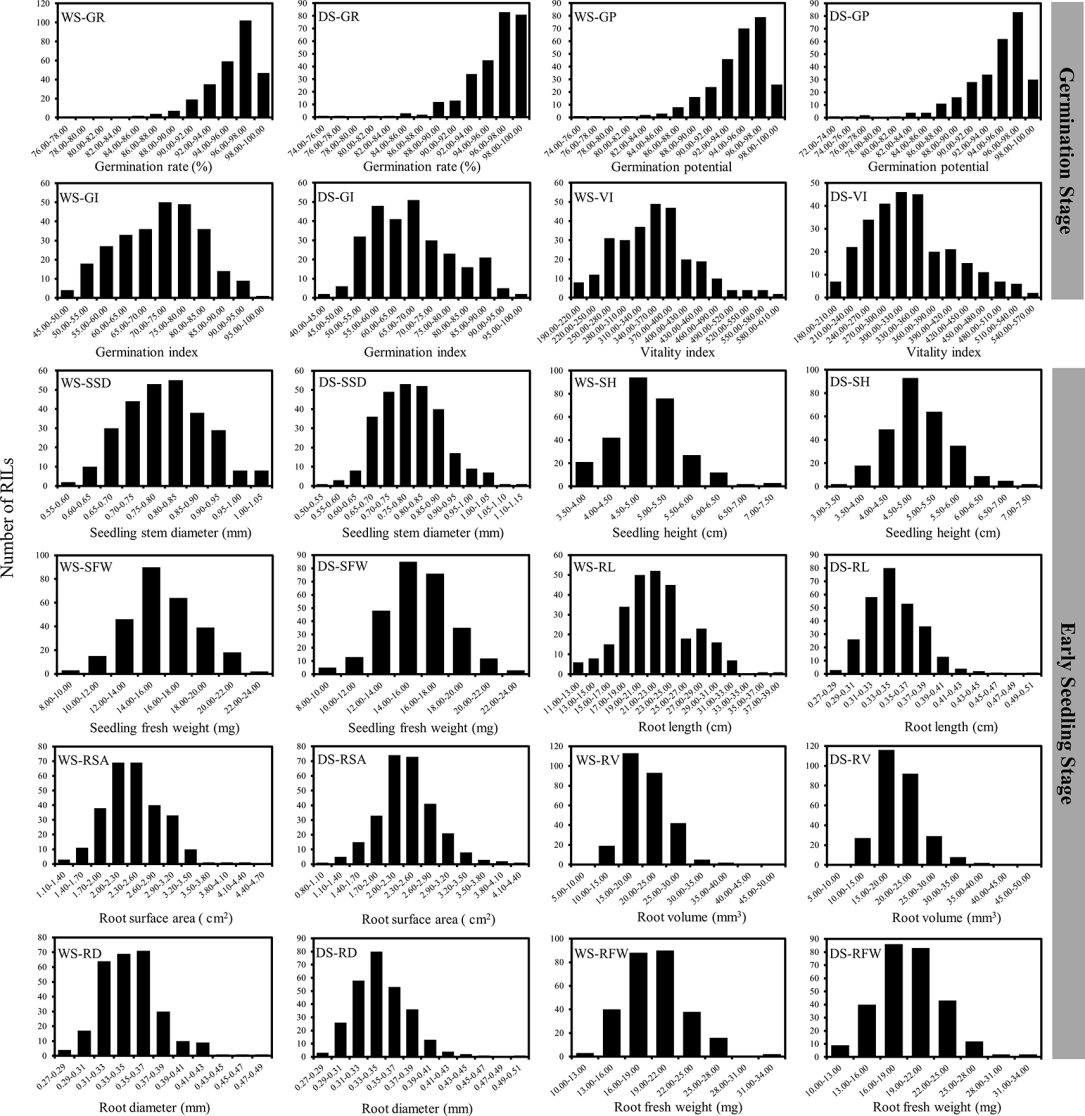
Frequency distribution of 12 traits in the RIL population.

As shown in **Table 1**, the differences between the two parents are obvious, especially the eight traits related to early seedling vigor. As for RILs population, all 12 traits varied widely among the population (*CV* = 3.050∼23.601% in WS, and 3.786∼24.605% in DS). Except GP and GR, all other traits showed high variation for both cropping seasons. Additionally, continuous single-peak pattern distributions were observed for all 12 investigated traits, which is consistent with quantitative traits controlled by multiple genes (**Table 1** and **Figure 1**). We also analyzed the broad-sense heritability across two environments. Heritabilities for eight traits related to early seedling vigor had a relatively high heritability of 96.770-98.570%. In contrast, four traits related to seed germination had a relatively low heritability of 48.230-86.180% (**Table 1**). Analysis of variance for the 12 traits was performed to detect the sources of phenotypic variation. Results showed that the phenotypic variances of eight traits related to early seedling vigor were significantly influenced by genetic factor; however, the phenotypic variances of four traits related to seed germination were significantly influenced by both genetic and genotype-by-environment interaction factors (**Supplementary Figure S1**).

**Table 1:** Phenotypic performance of germination and early seedlings across two cropping seasons.

Traits^a^	Environment^b^	Parents^c^		RIL population					Heritability^e^ (%)
		CHA-1	H335	Mean ± SD	Range	Skewness	Kurtosis	*CV* ^d^(%)	
GR (%)	WS	96.787 ± 1.628	95.292 ± 1.794	95.487 ± 2.913	79.184-100.000	-1.530	3.975	3.050	48.230
	DS	99.366 ± 0.669	98.991 ± 0.908	95.639 ± 3.620	75.677-100.000	-2.067	6.373	3.786	
GP	WS	95.434 ± 3.581	94.387 ± 2.301	94.356 ± 3.465	77.355-100.000	-1.389	2.903	3.673	49.920
	DS	99.143 ± 1.031	98.775 ± 0.557	94.069 ± 4.284	72.842-100.000	-1.768	4.539	4.554	
GI	WS	90.777 ± 2.383	67.965 ± 13.272**	71.413 ± 10.604	46.632-96.249	-0.134	-0.708	14.849	75.310
	DS	90.042 ± 4.249	74.044 ± 2.581**	66.933 ± 11.349	44.286-96.808	0.473	-0.493	16.956	
VI	WS	371.789 ± 9.759	320.926 ± 62.671	355.131 ± 78.053	193.602-603.966	0.482	0.295	21.979	86.180
	DS	376.431 ± 17.764	361.531 ± 12.601	332.896 ± 78.321	186.461-547.495	0.615	-0.145	23.527	
SSD (mm)	WS	0.657 ± 0.025	0.813 ± 0.016**	0.803 ± 0.096	0.552-1.026	0.149	-0.327	11.917	97.230
	DS	0.663 ± 0.010	0.823 ± 0.045**	0.796 ± 0.096	0.547-1.137	0.422	0.244	12.118	
SH (cm)	WS	4.096 ± 0.336	4.722 ± 0.331**	4.963 ± 0.653	3.535-7.179	0.501	0.638	13.150	98.570
	DS	4.124 ± 0.231	4.776 ± 0.270*	4.927 ± 0.663	3.427-7.147	0.493	0.531	13.464	
SFW (mg)	WS	11.369 ± 1.335	17.125 ± 0.983**	15.901 ± 2.584	9.425-23.631	0.234	-0.152	16.248	98.470
	DS	11.658 ± 0.508	16.383 ± 1.255**	15.748 ± 2.561	8.975-23.225	0.165	0.143	16.260	
RL (cm)	WS	13.157 ± 2.004	27.076 ± 3.332**	22.342 ± 4.617	11.421-39.221	0.410	0.405	20.665	97.160
	DS	14.076 ± 1.697	28.751 ± 2.919**	21.852 ± 4.616	10.338-36.451	0.246	0.092	21.122	
RSA (cm^2^)	WS	1.396 ± 0.337	2.997 ± 0.126**	2.420 ± 0.483	1.128-4.765	0.647	1.823	19.947	97.610
	DS	1.549 ± 0.297	3.167 ± 0.295**	2.362 ± 0.485	1.078-4.384	0.481	1.205	20.520	
RV (mm^3^)	WS	11.813 ± 4.264	27.438 ± 3.225**	21.052 ± 4.968	8.900-48.813	1.314	4.467	23.601	97.830
	DS	13.583 ± 3.563	27.792 ± 2.482**	20.489 ± 5.041	8.600-46.875	1.270	3.847	24.605	
RD (mm)	WS	0.337 ± 0.011	0.362 ± 0.006	0.347 ± 0.031	0.274-0.480	0.781	1.516	8.811	96.770
	DS	0.348 ± 0.027	0.351 ± 0.008	0.346 ± 0.031	0.274-0.492	0.903	1.966	9.085	
RFW (mg)	WS	13.175 ± 3.542	25.869 ± 1.558**	19.295 ± 3.628	10.925-32.513	0.416	0.470	18.802	98.050
	DS	14.483 ± 2.582	25.896 ± 0.683**	19.436 ± 3.494	11.913-33.394	0.667	0.786	17.977	

a Trait, GR, germination rate; GP, germination potential; GI, germination index; VI, vitality index; SSD, shoot stem diameter; SH, shoot height; SFW, shoot fresh weight; RL, root length; RV, root volume; RD, root diameter.

b Environment: WS is the wet season in 2017; DS is the dry season in 2017.

c Parent refers to the mean ± standard deviation (SD) of the parents, * and ** indicates significance at the levels of 0.05 and 0.01, respectively.

d CV, coefficient of variation.

e Heritability (%), broad-sense heritability in a single environment.

The intercorrelations of phenotypic values all pairs of the 12 traits are presented in **Table 2**. There were no strong correlations between traits related to seed germination and early seedling vigor. With respect to the four traits related to germination, there were strong correlations between GR and GP and between the GI and the VI. Among the shoot traits, the strongest positive correlation between lines occurred for SH and SFW. Among the root traits, the strongest positive correlation between lines occurred for RV and RSA.

**Table 2:** Pearson correlation matrix of 12 traits in the RIL population.

	Trait	GR	GP	GI	VI	SSD	SH	SFW	RL	RSA	RV	RD	RFW	
WS	GR		0.971**	0.229**	0.140*	-0.043	-0.051	-0.047	-0.081	-0.031	0.017	0.117	-0.007	DS
	GP	0.951**		0.320**	0.215**	-0.036	-0.034	-0.018	-0.075	-0.020	0.030	0.131*	0.014	
	GI	0.214**	0.347**		0.840**	0.068	0.204**	0.204**	0.049	0.118	0.157**	0.166**	0.172**	
	VI	0.109	0.215**	0.810**		0.112	0.669**	0.566**	0.280**	0.398**	0.434**	0.245**	0.416**	
	SSD	-0.138*	-0.085	0.158**	0.184**		0.148*	0.486**	0.381**	0.392**	0.322**	-0.013	0.436**	
	SH	-0.060	-0.033	0.226**	0.739**	0.135*		0.817**	0.497**	0.611**	0.610**	0.204**	0.561**	
	SFW	-0.084	-0.038	0.256**	0.646**	0.525**	0.796**		0.564**	0.696**	0.688**	0.236**	0.747**	
	RL	-0.064	-0.046	0.121*	0.388**	0.389**	0.527**	0.596**		0.896**	0.642**	-0.298**	0.701**	
	RSA	-0.029	-0.001	0.188**	0.502**	0.402**	0.637**	0.716**	0.900**		0.914**	0.147*	0.845**	
	RV	0.006	0.038	0.215**	0.518**	0.338**	0.624**	0.695**	0.649**	0.915**		0.525**	0.821**	
	RD	0.075	0.093	0.131*	0.202**	0.016	0.178**	0.210**	-0.305**	0.130*	0.510**		0.238**	
	RFW	-0.085	-0.045	0.249**	0.507**	0.461**	0.575**	0.758**	0.732**	0.840**	0.789**	0.162**		

*, ** significant at the 0.05 and 0.01 levels, respectively.

### Additive QTL Mapping in the RIL Population

**Figure 2:**
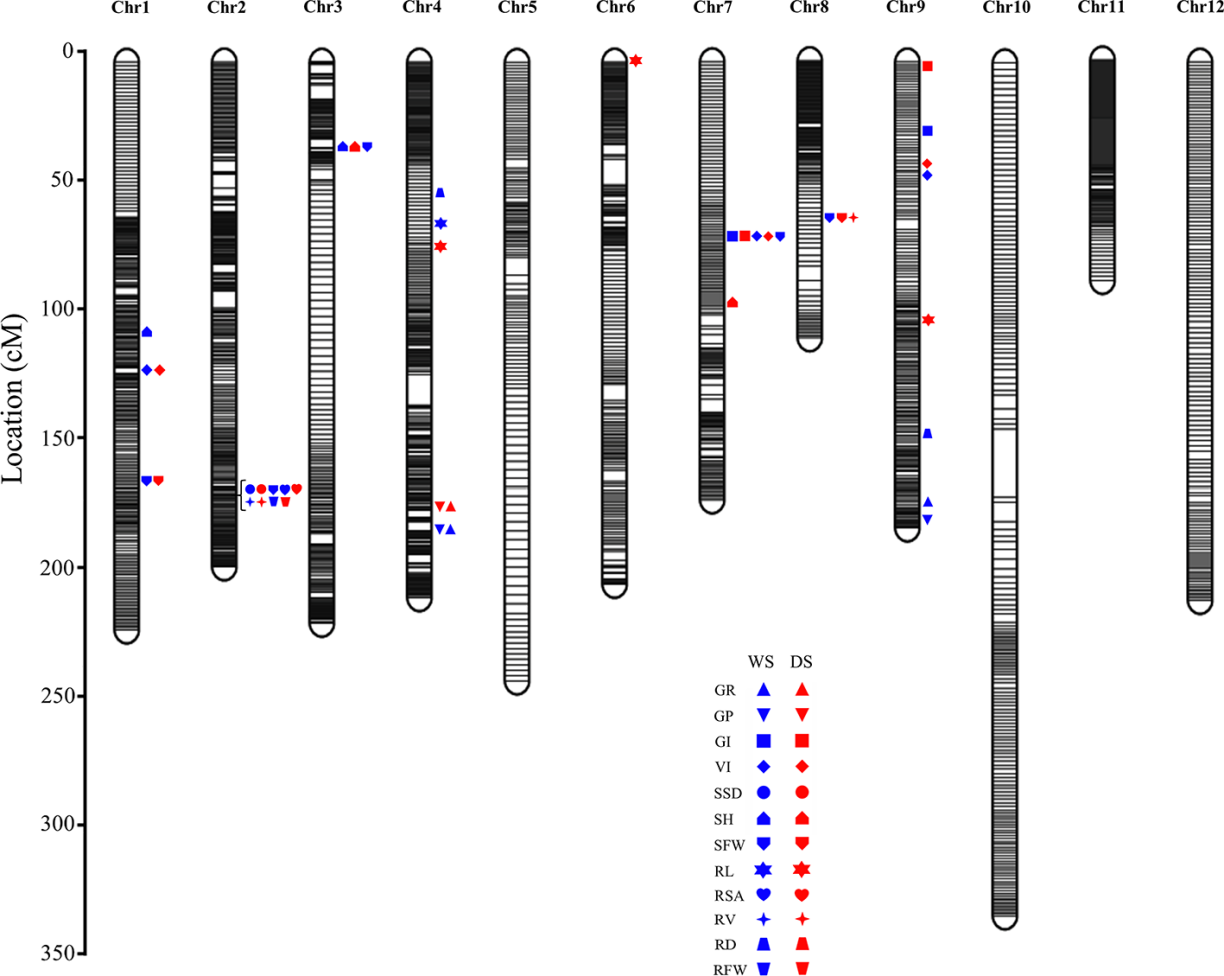
All additive QTL positions on the high-density map. The blue patterns represent the QTLs detected during the WS; the red patterns represent the QTLs detected during the DS. The text associated with different shapes represent abbreviations of different phenotypes.

Via the GBS approach, 100,307 biallelic homozygous SNPs were validated for the determination of recombinant events. Via the sliding window approach, a high-density bin map with an average physical length of 149.38 kb was obtained. This map contained 2498 bin markers that were evenly distributed across the 12 chromosomes. The number of markers on each chromosome ranged from 138 to 316. Via the inclusive composite interval mapping method, a total of 42 additive QTLs were detected by IciMapping v4.1 software with the use of a high-density map across two environments (23 for the WS and 19 for the DS). The phenotypic variation explained by these QTLs ranged from 0.50–27.43%. Among these QTLs, 16 were detected for germination, and 26 were detected for early seedlings; in addition, they were distributed on chromosomes 1, 2, 3, 4, 6, 7, 8, and 9 (**Table 3**). The QTLs that overlapped based on physical position were classified as the same loci. A total of 22 loci were ultimately obtained (**Table 3** and **Figure 2**).

**Table 3:** Additive QTLs detected in this study.

Loci	QTL	Env^a^	Chr.^b^	Marker interval	Physical interval (bp)	LOD^c^	PVE(%)^d^	ADD^e^	Donor of positive allele^f^	Known loci
loci1	*qSH-1*	WS	1	Block855-Block858	18,967,988-19,038,122	3.03	4.96	-0.1419	0	
loci2	*qVI-1*	WS	1	Block951-Block952	20,621,649-20,655,694	4.54	5.88	-19.3173	0	*qGR-1,* Wang et al., 2010
	*qVI-1*	DS	1	Block951-Block952	20,621,649-20,655,694	3.24	4.20	-16.4009	0	
loci3	*qSFW-1*	WS	1	Block1458-Block1495	32,722,412-33,092,963	3.64	5.19	-0.0006	0	
	*qSFW-1*	DS	1	Block1458-Block1495	32,722,412-33,092,963	3.88	0.50	-0.0006	0	
loci4	*qSSD-2*	WS	2	Block3971-Block4003	27,337,313-28,098,469	6.26	11.76	0.0306	2	*qSDW2,* Han et al., 2007
	*qSSD-2*	DS	2	Block3971-Block4003	27,337,313-28,098,469	5.23	10.84	0.0284	2	
	*qSFW-2*	WS	2	Block3988-Block3998	27,829,823-27,932,432	4.66	6.75	0.0006	2	
	*qRSA-2*	WS	2	Block3971-Block4003	27,337,313-28,098,469	3.18	5.69	0.1107	2	
	*qRSA-2*	DS	2	Block3971-Block4003	27,337,313-28,098,469	2.99	4.92	0.1076	2	
	*qRV-2*	WS	2	Block3978-Block3979	27,528,422-27,649,977	2.87	4.73	0.0011	2	
	*qRV-2*	DS	2	Block3978-Block3979	27,528,422-27,649,977	3.58	5.27	0.0012	2	
	*qRFW-2*	WS	2	Block3978-Block3979	27,528,422-27,649,977	5.77	9.28	0.0011	2	
	*qRFW-2*	DS	2	Block3978-Block3979	27,528,422-27,649,977	4.95	8.08	0.0010	2	
loci5	*qSH-3*	WS	3	Block5273-Block5280	19,437,008-19,449,809	3.36	5.56	0.1504	2	
	*qSH-3*	DS	3	Block5273-Block5280	19,437,008-19,449,809	2.71	4.77	0.1346	2	
	*qSFW-3*	WS	3	Block5273-Block5280	19,437,008-19,449,809	5.47	7.96	0.0007	2	
loci6	*qRD-4*	WS	4	Block6990-Block7002	11,554,467-12,117,492	3.26	4.85	-0.0069	0	
loci7	*qRL-4-1*	WS	4	Block7009-Block7015	12,294,020-12,542,201	3.61	5.86	1.1192	2	
loci8	*qRL-4-2*	DS	4	Block7073-Block7134	13,207,405-13,517,455	4.17	4.94	1.1657	2	
loci9	*qGR-4-1*	DS	4	Block7980-Block8015	27,396,341-28,476,977	3.51	5.93	-0.8684	0	*qGP-4,* Wang et al., 2010
	*qGP-4-1*	DS	4	Block7980-Block8015	27,396,341-28,476,977	3.40	6.75	-0.9855	0	
loci10	*qGR-4-2*	WS	4	Block8053-Block8084	29,926,217-30,392,121	3.19	5.78	-0.6466	0	
	*qGP-4-2*	WS	4	Block8053-Block8084	29,926,217-30,392,121	3.70	6.38	-0.8268	0	
loci11	*qRL-6*	DS	6	Block10156-Block10159	796,395-846,337	2.63	3.07	-0.9295	0	
loci12	*qGI-7*	WS	7	Block12037-Block12088	11,776,612-13,217,331	5.85	8.17	3.1786	2	
	*qGI-7*	DS	7	Block12037-Block12088	11,776,612-13,217,331	5.83	8.22	3.4060	2	
	*qVI-7*	WS	7	Block12037-Block12088	11,776,612-13,217,331	7.26	9.63	24.5986	2	
	*qVI-7*	DS	7	Block12037-Block12088	11,776,612-13,217,331	7.41	9.96	25.1284	2	
	*qSFW-7*	WS	7	Block12037-Block12088	11,776,612-13,217,331	2.59	3.65	0.0005	2	
loci13	*qSH-7*	DS	7	Block12131-Block12277	14,769,869-17,528,501	2.94	5.15	0.1385	2	
loci14	*qSFW-8*	WS	8	Block14052-Block14057	20,082,017-20,132,544	3.93	5.63	-0.0006	0	
	*qSFW-8*	DS	8	Block14052-Block14058	20,082,017-20,140,755	9.18	27.43	-0.0041	0	
	*qRV-8*	DS	8	Block14052-Block14057	20,082,017-20,132,544	2.82	4.14	-0.0011	0	
loci15	*qGI-9-1*	DS	9	Block14594-Block14626	1,081,787-1,914,120	2.79	3.85	2.3315	2	
loci16	*qGI-9-2*	WS	9	Block15150-Block15163	9,238,348-9,603,614	3.16	4.31	2.3156	2	
loci17	*qVI-9-1*	DS	9	Block15318-Block15321	11,394,951-11,459,454	3.20	4.15	16.2650	2	
loci18	*qVI-9-2*	WS	9	Block15394-Block15400	11,812,305-11,857,467	2.82	3.60	15.0605	2	
loci19	*qRL-9*	DS	9	Block15759-Block15762	17,901,269-18,025,376	3.39	3.98	-1.0451	0	
loci20	*qRD-9*	WS	9	Block15929-Block15931	20,396,914-20,444,283	3.81	5.71	0.0074	2	
loci21	*qGR-9*	WS	9	Block16052-Block16053	21,755,498-21,895,114	2.54	4.63	0.5783	2	
loci22	*qGP-9*	WS	9	Block16059-Block16061	22,015,280-22,077,000	2.57	4.42	0.6892	2	

a Environment: WS represents the wet season in 2017; DS represents the dry season in 2017.

b Chr, chromosome.

c LOD, logarithm of odds.

d ADD, additive effect; positive values indicate effects of alleles from regions with marker type 2.

e PVE (%), phenotypic variation explained (%).

f Donor of positive allele; the source of increasing allele effects was an allele from regions with either marker type 2 or marker type 0.

### Identification of the Interactions Between Additive QTLs and the Environment

To identify the interactions between additive QTLs and the environment, joint analyses of multienvironment phenotypic values for the 12 traits during two cropping seasons were conducted *via* the "MET" approach of QTL IciMapping. Forty-six QTLs (32 loci) were identified (**Supplementary Table S2**). The phenotypic variation explained by these QTLs ranged from 2.19-12.18%. These QTLs were distributed across all chromosomes except 11 and 12. The results were then compared with those of the additive QTL mapping in a single environment. With the exception of loci 3 (*qSFW-1*), all additive locus mapping within a single environment could be identified by multienvironment QTL joint mapping. In addition, among the 17 QTLs related to germination, six QTLs had a higher contribution rate of interactive effects than of additive effects. However, among the 29 QTLs related to early seedling growth, only one QTL had a higher contribution rate of interactive effects than of additive effects. This finding indicates that the interaction between QTLs and the environment is one of the main genetic factors for germination traits. Notably, these results were also supported by the heritability analysis.

### Quality of QTL Mapping

Segregation distortion is commonly observed in populations (Xu, 2008). We mapped the regions of segregation distortion. Interval mapping for SDLs revealed four significant intervals that were skewed toward either parent (**Table 4**). Notably, there were no significant SDLs colocalized to the QTLs detected in this study. Therefore, the QTLs mapped in this study were not affected by segregation distortion within chromosomal regions.

**Table 4:** Interval mapping of SDLs within the RIL population.

SDL	Marker interval	Physical interval (bp)	LOD	Segregation ratio	
				Marker type 0	Marker type 2
*sdl-2*	Block3113-Block3127	16,096,502-16,558,187	3.58	1	0.6082
*sdl-3-1*	Block4633-Block4698	3,322,276-4,197,129	4.19	1	0.5831
*sdl-3-2*	Block6055-Block6075	32,024,821-32,543,113	3.53	1	0.6104
*sdl-8*	Block14146-Block14417	24,529,237-26,529,908	4.26	1	0.5805

To further verify the accuracy of our results, we compared the QTLs in this study with previously reported QTLs. We discovered three loci that have been reported previously (**Table 3**). Loci 2, which is associated with the VI, was identified in the genomic interval of *qGR-1*, which is related to the GR at 3 days (Wang et al., 2010). Loci 4, which is associated with SSD, SFW, RSA, RV and RFW, was identified in the genomic interval of *qSDW2*, which is related to seedling dry weight under 12°C cold water irrigation (Han et al., 2007). Loci 9, which is associated with GP and GR, was identified in the genomic interval of *qGP-4*, which is related to the final germination percentage at 10 days (Wang et al., 2010). Overall, our QTLs mapped by this high-density bin map are creditable.

### Identification of Stable Additive Loci

**Figure 3:**
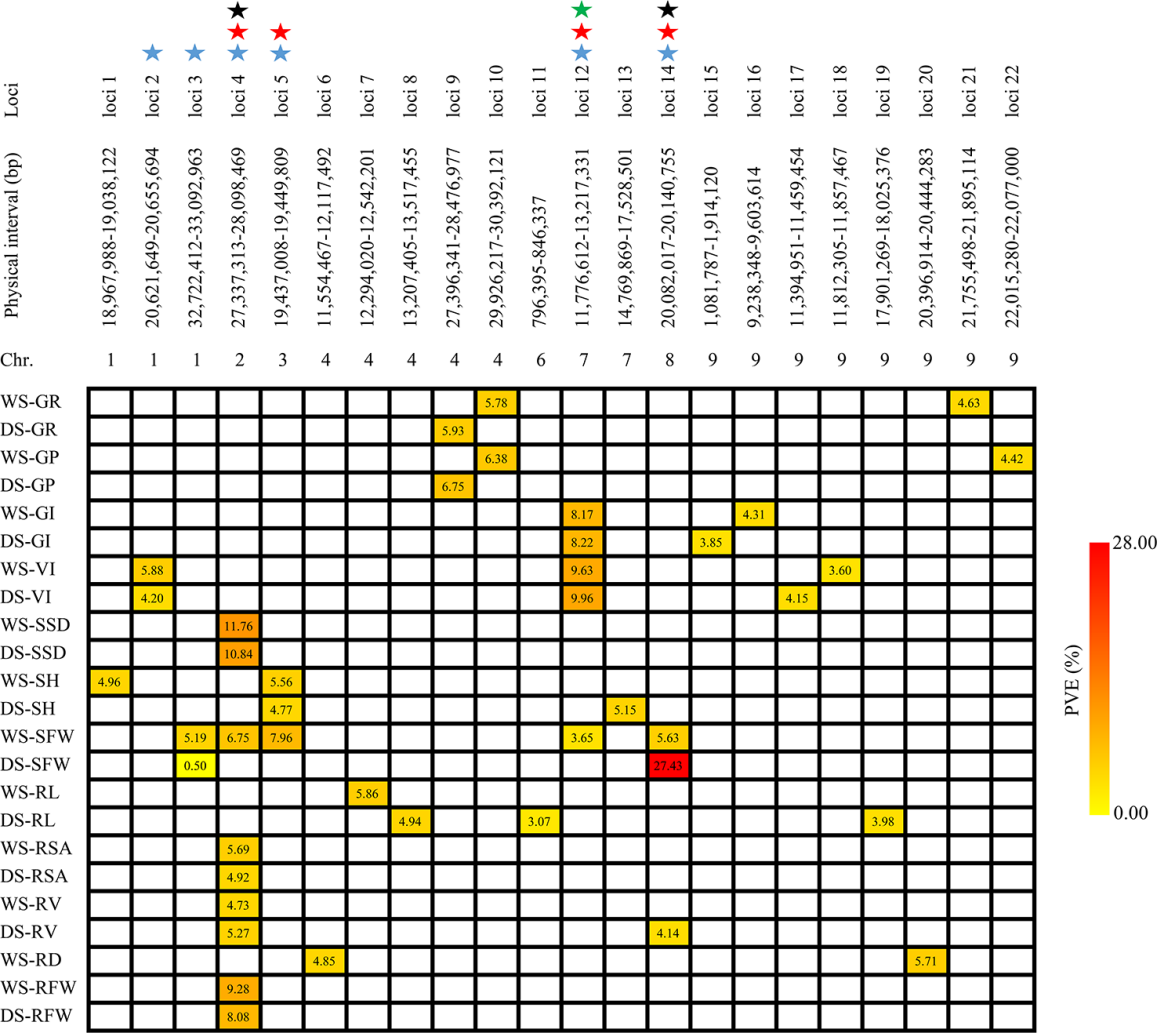
Summary of 22 additive loci associated with 12 traits. The different colors shown in the legend correspond to different levels of phenotypic variation explained. The blue star means detected in both seasons, the red star represents the simultaneous influence of multiple traits, the black star represents the trait that affects both shoots and roots for early seedling growth, and the green star indicates that both seed germination and early seedling growth are affected.

Although joint analysis can detect more QTLs. However, due to the environmental effect, the QTLs were identified separately in each environment, and the loci detected in common across multiple environments were considered as stable and high credible QTL. In our study, six of the 22 additive loci, that is, loci 2, 3, 4, 5, 12, and 14, were repeatedly detected across the two seasons (**Table 3** and **Figure 3**). Because loci 3 (*qSFW-1*) was not detected by joint analyses; therefore, loci 2, 4, 5, 12, and 14 were identified as stable and highly credible. In addition, four of the five stable additive loci exhibited clear pleiotropic effects that were associated with multiple traits: specifically, loci 4 was associated with SSD, SFW, RSA, RV, and RFW; Loci 5 was associated with both SH and SFW; Loci 12 was associated with the GI, VI, and SFW; and Loci 14 was associated with both SFW and RV. Notably, loci 4 and 14 affect both shoot and root traits of early seedlings, and loci 12 was detected for both germination and early seedling growth.

### Validation of the Consequences of the Five Stable Loci

To further clarify the effects of the five stable QTLs, we summarized the phenotypic differences between the two alleles of each locus in the RIL population (**Table 5**). We first divided the individuals of the RIL population into the marker 2 type and marker 0 type of each identified locus and then compared the differences between the two genotypes for the corresponding traits. The results showed that the corresponding traits’ mean phenotypic values of RILs harboring excellent alleles were significantly greater than those of RILs harboring nonexcellent alleles (*p* < 0.01). In other words, these five stable loci could efficiently increase the corresponding traits.

**Table 5:** Summary of the phenotypic effects of five stable loci.

Loci	Traits	QTL^a^	Env^b^	Number of RILs of Marker type 0	Number of RILs of Marker type 2	Donor of positive allele	Phenotypic value		Difference^c^
							Marker type 0	Marker type 2	
loci2	VI	*qVI-1*	WS	148	113	0	372.335 ± 79.753	331.208 ± 67.846	41.127**
		*qVI-1*	DS				347.783 ± 82.167	310.301 ± 69.334	37.482**
loci4	SSD (mm)	*qSSD-2*	WS	140	106	2	0.775 ± 0.096	0.831 ± 0.087	0.056**
		*qSSD-2*	DS				0.770 ± 0.097	0.821 ± 0.086	0.051**
	SFW (mg)	*qSFW-2*	WS				15.144 ± 2.384	16.570 ± 2.568	1.426**
		*-*	DS				15.082 ± 2.459	16.350 ± 2.486	1.269**
	RSA (cm^2^)	*qRSA-2*	WS				2.314 ± 0.445	2.530 ± 0.489	0.216**
		*qRSA-2*	DS				2.260 ± 0.468	2.461 ± 0.478	0.201**
	RV (mm^3^)	*qRV-2*	WS				20.033 ± 4.492	22.009 ± 4.977	1.976**
		*qRV-2*	DS				19.446 ± 4.563	21.535 ± 5.231	2.089**
	RFW (mg)	*qRFW-2*	WS				18.379 ± 3.073	20.566 ± 3.627	2.188**
		*qRFW-2*	DS				18.303 ± 3.302	20.343 ± 3.698	2.040**
loci5	SH (cm)	*qSH-3*	WS	148	112	2	4.837 ± 0.617	5.132 ± 0.653	0.296**
		*qSH-3*	DS				4.780 ± 0.622	5.089 ± 0.675	0.290**
	SFW (mg)	*qSFW-3*	WS				15.320 ± 2.482	16.718 ± 2.554	1.398**
		-	DS				15.162 ± 2.511	16.508 ± 2.473	1.346**
loci12	GI	*qGI-7*	WS	126	128	2	68.541 ± 10.321	74.078 ± 10.096	5.538**
		*qGI-7*	DS				63.782 ± 10.563	70.301 ± 11.449	6.519**
	VI	*qVI-7*	WS				332.139 ± 70.158	375.168 ± 79.324	43.029**
		*qVI-7*	DS				309.409 ± 71.760	355.868 ± 79.523	46.459**
	SFW (mg)	*qSFW-7*	WS				15.272 ± 2.453	16.492 ± 2.573	1.220**
		-	DS				14.986 ± 2.455	16.409 ± 2.521	1.423**
loci14	SFW (mg)	*qSFW-8*	WS	136	108	0	16.257 ± 2.792	15.331 ± 2.304	0.927**
		*qSFW-8*	DS				16.068 ± 2.669	15.223 ± 2.420	0.845**
	RV(mm3)	*-*	WS				21.627 ± 5.414	20.014 ± 4.238	1.613**
		*qRV-8*	DS				21.234 ± 5.152	19.287 ± 4.475	1.947**

a QTL,"-" indicates that no QTL was detected during the season.

b Environment: WS represents the wet season in 2017; DS represents the dry season in 2017.

c Phenotype of an elite allele minus that of a nonelite allele; **significant at the 0.01 level.

### Screening Materials That Pyramid the Elite Alleles of the Five Stable Loci

**Figure 4:**
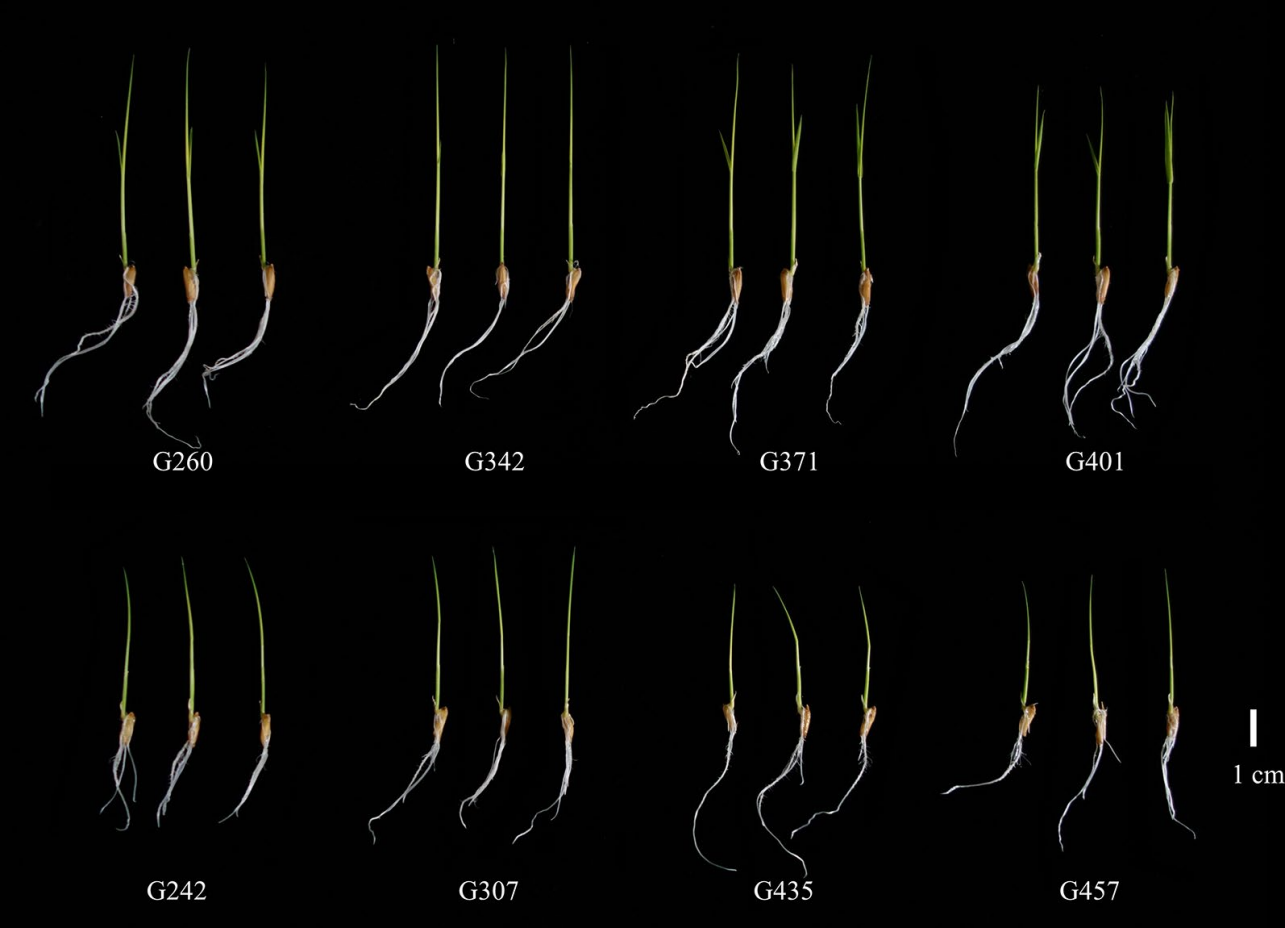
The seedling growth phenotypes of four RILs (upper panel) that pyramided the excellent alleles and four RILs (lower panel) that pyramided the non-excellent alleles of the five stable genetic loci.

In this population, we identified ten RILs that pyramided the excellent alleles of five stable genetic loci and eleven RILs that pyramided the non-excellent alleles of five stable genetic loci. Although, the five stable loci were associated with only eight traits (excluding GR, GP, RL and RD). However, we found that most phenotypic values of the ten RILs were greater than the average values, and the eleven RILs are the opposite (**Supplementary Table S3**). This result also indicated that the pyramiding of excellent alleles could improve the phenotypic value. By combining the phenotypes of both seasons, we further selected four RILs (G260, G342, G371, and G401) that had more excellent phenotypic values from the ten RILs that pyramided the excellent alleles; and also selected four RILs (G242, G307, G435, and G457) that had non excellent phenotypic values from the eleven RILs that pyramided the non-excellent alleles. These two types of RILs showed big differences in seed germination and early seedling growth (**Supplementary Figure S2**, **Table S3** and **Figure 4**). In a word, the four RILs (G260, G342, G371, and G401) could serve as donor parents of favorable alleles in the breeding process.

### Identification of Candidate Genes From the Five Stable Loci

**Figure 5:**
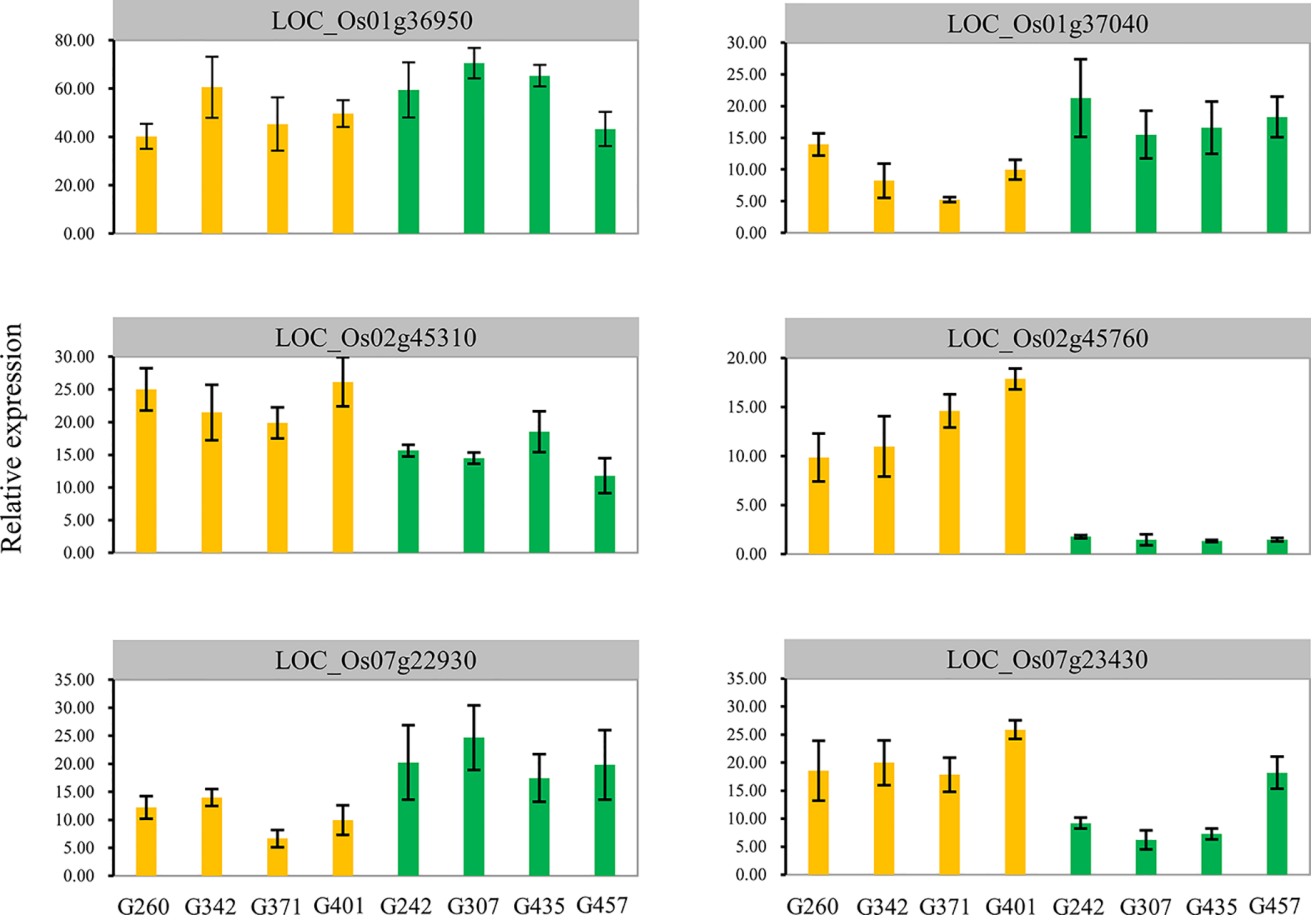
Expression analysis of candidate genes. The expression levels of the six candidate genes in tissues (seeds + seedlings) of germinated three-day-old seeds and seedlings were measured using quantitative RT-PCR. The x-axis lists the eight genotypes whose phenotypes contrast (four excellent RILs: G260, G342, G371, and G401; four nonexcellent RILs: G242, G307, G435, and G457). The error bars correspond to the standard errors (n = 3).

In the interval and flanking regions of the five stable loci, 351 genes were identified (**Supplementary Table S4**). We predicted 20 candidate genes based on a previously reported expression profile of seed germination (Howell et al., 2008) and gene annotation data (**Table 6**). To narrow down the candidate genes, we further validated the expression levels of these 20 genes using the differences in qRT-PCR results between H335 and CHA-1 germinated seeds from the third day of the experiment. The results showed that the expression levels of LOC_Os01g36950, LOC_Os01g37040, LOC_Os02g45310, LOC_Os02g45760, LOC_Os07g22930, and LOC_Os07g23430 were significantly different (**Table 6**). We also examined expression patterns of these six candidate genes in the four excellent RILs and four nonexcellent RILs. Surprisingly, the expression patterns of LOC_Os01g37040, LOC_Os02g45310, LOC_Os02g45760, LOC_Os07g22930, and LOC_Os07g23430 can clearly distinguish the two groups of RILs, and are similar to the expression patterns of the parents (**Table 6** and **Figure 5**).

**Table 6:** Twenty candidate genes were predicted for five stable loci.

Stable loci	Gene ID	Relative expression			Description
		H335	CHA-1	Sig.	
loci2	LOC_Os01g36950	55.89 ± 2.26	73.23 ± 1.98**	yes	N-rich protein, putative, expressed
	LOC_Os01g36970	1.00 ± 0.03	0.64 ± 0.26	no	expressed protein
	LOC_Os01g37020	13.89 ± 1.58	11.97 ± 3.16	no	carboxyl-terminal peptidase, putative, expressed
	LOC_Os01g37040	8.18 ± 0.98	15.83 ± 1.26**	yes	retrotransposon protein, putative, Ty1-copia subclass, expressed
loci4	LOC_Os02g45310	21.12 ± 0.52	13.96 ± 0.84**	yes	dehydration response related protein, putative, expressed
	LOC_Os02g45390	3.43 ± 0.58	2.32 ± 0.45	no	RING-H2 finger protein, putative, expressed
	LOC_Os02g45690	4.45 ± 0.24	6.51 ± 1.71	no	uncharacterized mscS family protein, putative, expressed
	LOC_Os02g45760	14.92 ± 3.08	3.92 ± 1.05**	yes	chitin-inducible gibberellin-responsive protein 2, putative, expressed
	LOC_Os02g45890	2.38 ± 0.29	1.91 ± 1.07	no	sulfotransferase domain containing protein, expressed
	LOC_Os02g45940	24.00 ± 2.62	28.65 ± 4.13	no	Core histone H2A/H2B/H3/H4 domain containing protein, putative, expressed
	LOC_Os02g46030	1.57 ± 0.25	1.16 ± 0.30	no	MYB family transcription factor, putative, expressed
loci5	LOC_Os03g34040	7.30 ± 0.62	7.97 ± 1.00	no	ribosomal protein, putative, expressed
loci12	LOC_Os07g22400	6.83 ± 0.53	5.41 ± 0.22*	yes	POLA3 - Putative DNA polymerase alpha complex subunit, expressed
	LOC_Os07g22580	7.05 ± 1.54	6.51 ± 1.93	no	rhoGAP domain containing protein, expressed
	LOC_Os07g22710	32.00 ± 1.54	30.88 ± 5.13	no	CAMK_CAMK_like.32 - CAMK includes calcium/calmodulin depedent protein kinases, expressed
	LOC_Os07g22930	10.30 ± 3.10	28.07 ± 10.39**	yes	starch synthase, putative, expressed
	LOC_Os07g23120	8.79 ± 1.60	5.41 ± 1.71	no	expressed protein
	LOC_Os07g23430	26.03 ± 2.81	13.20 ± 0.22**	yes	fatty acid desaturase, putative, expressed
loci14	LOC_Os08g32500	4.95 ± 0.52	4.48 ± 1.47	no	nucleobase-ascorbate transporter, putative, expressed
	LOC_Os08g32520	8.74 ± 2.37	6.74 ± 0.71	no	expressed protein

*and ** indicates significance at the levels of 0.05 and 0.01, respectively.

## Discussion

Rapid, uniform germination and vigorous seedling growth are essential for the direct seeding of rice (Mahender et al., 2015). In flooded conditions after direct seeding, a vigorous root system can help plants quickly anchor in the soil, reducing the amount of unevenness caused by floating or dead seedlings, thus laying the foundation for future yield. Therefore, root phenotypes are important phenotypic indicators. However, most researchers in previous reports investigating seedling roots adopted indexes related to only the maximum RL and root weight (Zhang et al., 2005; Diwan et al., 2013; Dang et al., 2014; Zhang et al., 2017; Singh et al., 2017). In this study, we collected total RL, RSA, RV, RD and RFW data, which can not only provide in-depth seedling roots detail of but also improve the efficiency of mapping. Eight root-related loci were ultimately identified, seven of which are novel (**Table 3** and **Figure 3**).

Strong interactions between genotypes and environments have been observed for some important agronomic traits, such as grain yield, heading date, and plant height (Zhuang et al., 1997; Li et al., 2003). Most QTLs related to these traits are sensitive to the conditions imposed and are environment specific. However, a few QTLs are relatively stable and are always detected in different experiments. In double-cropping rice areas in South China, the environmental differences between the WS and DS are substantial. Therefore, it is necessary to clarify the influence of environmental factors on the process of seed vigor formation. Although we investigated the rice heading date and individual plants with consistent maturity were selected, healthy seeds were selected for experiments to help minimize the impact of environmental factors. However, we still observed significant interactions between the environment and QTLs. Notably, we found that the interaction between QTLs related to seed germination and the environment was stronger than that between QTLs related to early seedling vigor and the environment (**Supplementary Table S2**). This finding may be due to the pregermination selection of the same radicle or germ for experiments. Thus, some environmental factors are additionally excluded. Overall, we identified five stable loci that are ideal targets for direct seeding and rice breeding.

QTL mapping resolution depends on marker density and the size of the confidence interval of QTLs (Visscher et al., 1996; Da et al., 2000). Generally, a gain in information as a result of increased marker density results in smaller intervals; therefore, the application of additional markers is an effective way to increase QTL mapping resolution (Liu et al., 2008). In the present study, a high-density bin map for the RIL population was used for QTL mapping in rice. Compared with previous QTLs associated with rice seed germination and early seedling growth, the QTL interval size in this study was significantly narrow, and the physical interval of the five stable loci is only approximately 170 kb on average, which contributes to the fine mapping of these loci. In the interval and flanking regions of the five stable loci, 351 genes were identified (**Supplementary Table S4**). We predicted 20 candidate genes based on a previously reported expression profile of seed germination (Howell et al., 2008) and gene annotation data, we also further identified five promising candidate genes by qRT-PCR (**Table 6** and **Figure 5**). These genes provide a foundation for understanding the mechanism of rice germination and early seedling growth.

Among the five stable loci, loci 2 colocated with the previously reported *qGR-1* (Wang et al., 2010), and loci 4 colocated with *qSDW2* (Han et al., 2007). Notably, using this population, we also performed QTL mapping for anaerobic germination (data unpublished) and found that loci 12 also controlled anaerobic germination. Therefore, we inferred that loci 12 is not exclusively induced by specific environments and probably is a genetic loci necessary for the process of rice seed germination under different stresses. In a word, these results further demonstrate the reliability of our results. In addition, loci 4, loci 5, loci 12 and loci 14 exhibited clear pleiotropy. In particular, loci 4 was associated with five traits. Further study of these stable and pleiotropic loci will help to explain the complex genetic mechanisms underlying seed germination and early seedling growth.

Pyramiding QTLs using marker-assisted selection (MAS) is effective for achieving the desirable phenotypic level of a quantitative trait in plant breeding programs (Ashikari and Matsuoka, 2006; Nagai et al., 2012). However, the outcomes of pyramiding QTLs are not always as expected because of, for example, epistatic interactions between QTLs (Miedaner et al., 2006; Bovill et al., 2010). In this study, we detected several epistatic QTLs, namely, GR-, GP-, GR- and RV-related epistatic QTLs. Most pairs of interacting QTLs presented a small PVE, and only one pair of interacting QTLs had a PVE as high as 10.46% detected in the WS (**Supplementary Table S5**). Therefore, we believe that the epistatic effect between QTLs is not the main genetic factor for germination and early seedling growth. Fortunately, RILs have pyramided excellent alleles of the five stable genetic loci. Although these five stable loci did not encompass all QTLs, most phenotypes of these 10 RILs indeed improved. In addition, four of these loci exhibited obvious pleiotropic effects, which also increased the efficiency of pyramid breeding.

## Data Availability Statement

The raw data supporting the conclusions of this manuscript will be made available by the authors, without undue reservation, to any qualified researcher.

## Author Contributions

TG, ZC, and JY designed the project, and JY performed all the experiments and wrote the manuscript. GY, MY, LS, AX, DL, CH, DZ, YL, and HW assisted in conducting the experiments and analyzing the data. HW and ZC provided the direction for the study and the corrections of the manuscript. All authors read and approved the final manuscript.

## Funding

This research was supported by the Breeding New Varieties of Rice Suitable for Light and Simple Cultivation and Mechanized Production Project (No. 2017YFD0100104) of Ministry of Science and Technology of the People’s Republic of China, the National Key Technology Research and Development Program (No. 2016YFD102102) of Ministry of Science and Technology of the People’s Republic of China, the Science and Technology Project of Guangdong Province (No. 2015B020231011) and the earmarked fund for Modern Agro-Industry Technology Research System of the Ministry of Agriculture and Rural Affairs of the People’s Republic of China (No. CARS-01-12).

## Conflict of Interest

The authors declare that the research was conducted in the absence of any commercial or financial relationships that could be construed as a potential conflict of interest.

## Abbreviations

GR, germination rate; GP, germination potential; GI, germination index; VI, vitality index; SSD, shoot stem diameter; SH, shoot height; SFW, shoot fresh weight; RL, root length; RSA, root surface area; RV, root volume; RD, root diameter; RFW, root fresh weight; RIL: recombinant inbred line; QTL: quantitative trait loci; DSR: directly seeded rice; WS: wet season; DS: dry season; GBS: genotyping-by-sequencing.
