# Supplementary figures and images for: Quantitative Trait Locus Analysis of Seed Germination and Early Seedling Growth in Rice
Source: Front Plant Sci. 2019 Dec 13;10:1582. doi: 10.3389/fpls.2019.01582 (PMC6923285; doi:10.3389/fpls.2019.01582)

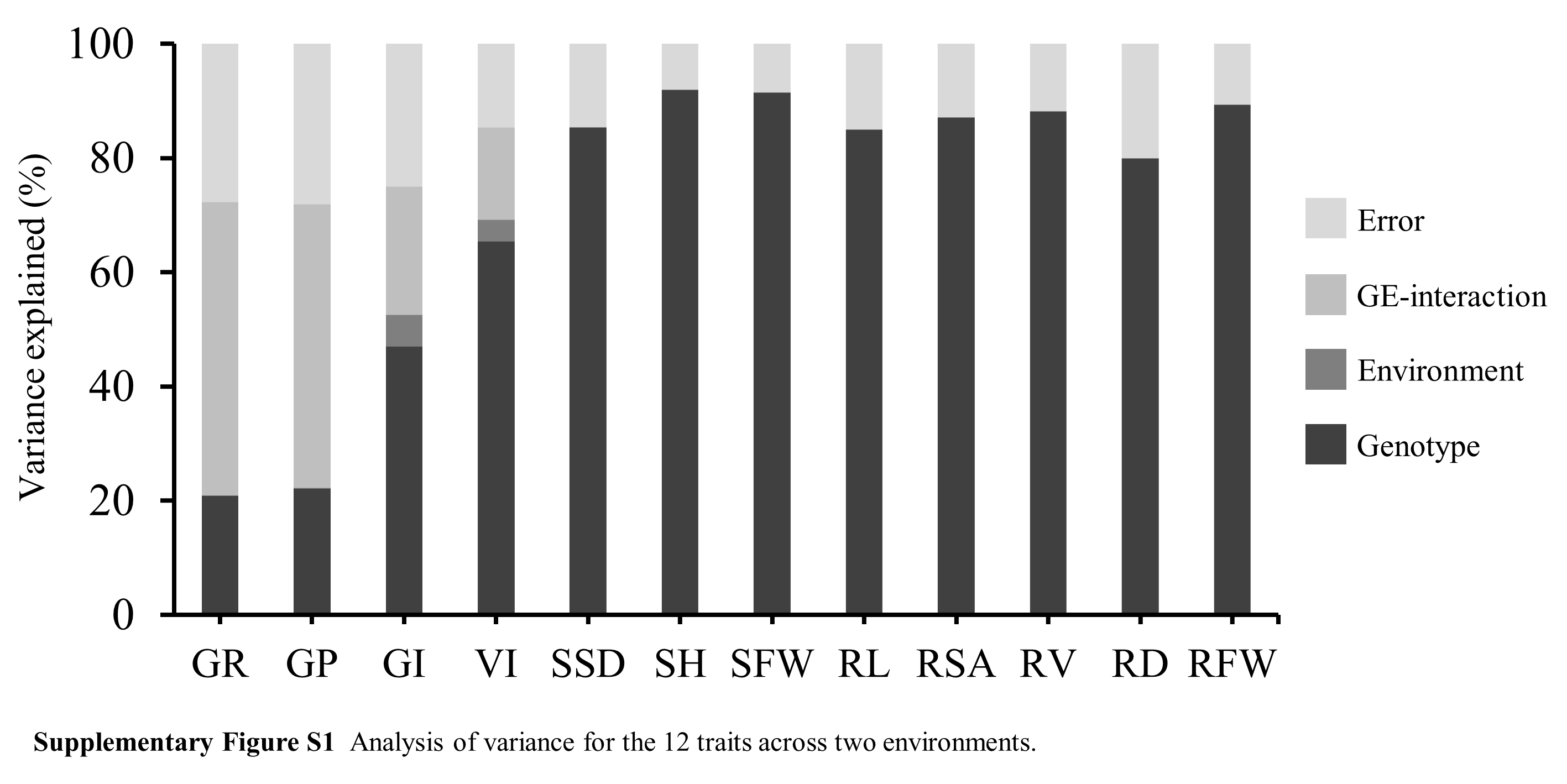

Supplement: Supplementary file 1 [file Image_1.tif]
